# Fructation *In Vivo*: Detrimental and Protective Effects of Fructose

**DOI:** 10.1155/2013/343914

**Published:** 2013-07-24

**Authors:** H. M. Semchyshyn

**Affiliations:** Department of Biochemistry and Biotechnology, Vassyl Stefanyk Precarpathian National University, 57 Shevchenko Street, Ivano-Frankivsk 76025, Ukraine

## Abstract

There is compelling evidence that long-term intake of excessive fructose can have deleterious side effects in different experimental models. However, the role of fructose *in vivo* remains controversial, since acute temporary application of fructose is found to protect yeast as well as animal tissues against exogenous oxidative stress. This review suggests the involvement of reactive carbonyl and oxygen species in both the cytotoxic and defensive effects of fructose. Potential mechanisms of the generation of reactive species by fructose in the nonenzymatic reactions, their implication in the detrimental and protective effects of fructose are discussed.

## 1. Introduction

Over the past decade, considerable scientific debate and controversy have arisen regarding the biological role of the reducing monosaccharides, and fructose in particular [1, 2]. Since many nutritionists believe that fructose is safer and healthier than glucose, fructose often is advocated as a glucose substitute by diabetes mellitus patients and a preferred sweetener for different population groups. At the same time, numerous epidemiological, clinical, and experimental studies demonstrate strong positive relationship between the intake of fructose and the development of metabolic disturbances [3–16]. Although consumption of fructose may have the adverse side effects and some authors state that there are no data showing a protective effect of fructose [3], it should also be mentioned that acute temporary application of fructose is found to be beneficial under some conditions [17–23]. 

Potential mechanisms underlying both detrimental and protective effects of fructose are under debate. Nonenzymatic reactions of fructose and higher production of reactive carbonyls (RCS) and oxygen species (ROS) compared with glucose are believed to be causative in negative effects of fructose [24–26]. However, ROS and RCS are found to play a dual role *in vivo*, which appears to be dose and time dependent [23, 27–32]. Therefore, we suggest the involvement of reactive species in both the cytotoxic and defensive effects of fructose. This review examines some of the potential mechanisms of ROS and RCS generation by the nonenzymatic reactions of fructose, their implication in the detrimental and defensive effects of fructose *in vivo*, and some of the differences between the long-term and short-term applications of fructose.

## 2. Involvement of Fructose in the Maillard Chemistry

The nonenzymatic reaction between amino acids and reducing monosaccharides was first described by Maillard a century ago [33, 34]. 40 years later, the Maillard reaction was recognized as one of the main reasons for the occurrence of the nonenzymatic food browning demonstrating an importance in food science [35, 36]. In late 1960s, the products of nonenzymatic glycosylation similar to the products of food browning were detected in human organism [37, 38]. It took several decades to realize the physiological significance of the reaction described by Maillard, which received renewed attention in biochemistry and medicine. The nonenzymatic glycosylation has been named “glycation” in order to differentiate it from the enzymatic glycosylation, an important posttranslation modification of proteins [39]. In 1980s, Monnier and Cerami postulated that glycation had a causative role in aging and age-related pathologies [40, 41]. Today, their theory called the “glycation hypothesis of aging” is at the origin of the growing interest in the field of *in vivo* glycation, aging, and age-related diseases. 

Glycation is a process in which various compounds, including RCS and advanced glycation end-products (AGEs), are produced [36, 42–45]. An increase in the RCS and AGEs steady-state levels may result in so-called carbonyl stress. The concept of “carbonyl stress” was introduced by Miyata and colleagues in late 1990s [46]. The authors have defined carbonyl stress as a situation “resulting from either increased oxidation of carbohydrates and lipids (oxidative stress) or inadequate detoxification or inactivation of reactive carbonyl compounds derived from both carbohydrates and lipids by oxidative and non-oxidative chemistry.” RCS are mainly known for their damaging effects. At the molecular level, RCS are found to modify the structure of proteins, nucleic acids, lipids, and carbohydrates. As a consequence, the loss of functions and even viability can occur at the cellular and organismal levels. These harmful effects of RCS are mainly linked to the initiation of glycation [36, 43, 47]. Therefore, a vicious cycle can be created* in vivo* when RCS serve as either the initiators or products of glycation. 

It should be noted that reactive carbonyls are commonly generated *in vivo* as metabolic products [36, 44, 45, 48]. For example, oxidation of such amino acids as threonine and glycine can lead to RCS formation under physiological conditions [49]. Different RCS can be generated *in vivo *by activated human phagocytes. It has been found that stimulated neutrophils employed the myeloperoxidase-H_2_O_2_-chloride system to produce *α*-hydroxy and *α*,*β*-unsaturated aldehydes from hydroxy amino acids in high yield [49]. 

Besides highly reactive RCS, carbohydrates are important glycating agents. In general, RCS may demonstrate 20,000-fold higher reactivity than some reducing monosaccharides [48]; however the latter are more abundant intra- and extracellular glycation agents. The contribution of carbohydrates to nonenzymatic processes has been extensively investigated over few last decades. This may be attributed to either beneficial or detrimental effects of reducing carbohydrates *in vivo*, and most studies in the field of glycation are focused on glucose (glucation). Fructose is another reducing monosaccharide, a common component of honey, fruit juice concentrates, table sugar, and high-fructose corn syrup. It has been excessively consumed in human diets over the last decades, despite the evidence implicating fructose in the development of metabolic and other disorders [3–6]. However, glycation by fructose (fructation) has not been as thoroughly investigated as that of glucose. 

**Figure 1:**
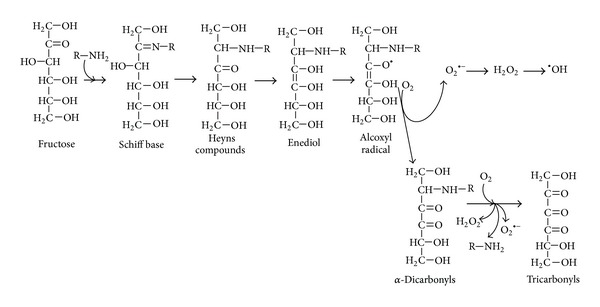
Suggested mechanism for the production of reactive carbonyl and oxygen species by the fructation.

The initial step of fructation is the covalent interaction between free carbonyl group of open-chain fructose and amino group of biomolecule, producing the Schiff base (Figure 1). The latter is an unstable compound that can be subjected to further isomerization (Heyns rearrangement) and form more stable Heyns adducts. The Heyns compounds as well as Amadori products derived from glucation are known as “early glycation products” or “fructosamines.” The fructose moiety of the Heyns products can undergo enolization followed by dehydration, oxidation, and/or fragmentation reactions, consequently producing a variety of RCS [36, 50–52]. 

RCS can also be formed due to enzymatic reactions of reducing carbohydrates (e.g., glucose or fructose). For example, polyol pathway may be associated with the production of glyoxal, methylglyoxal, glucosone, and 3-deoxyglucosone [49]. Effective steady-state concentration of such RCS metabolites as acetaldehyde, glyceraldehyde-3-phosphate, and dihydroxyacetone phosphate is typically low in the cell because of their rapid utilization by the next step of the pathway [49]. However, the concentration of such by-products of glycolysis, polyol pathway, or enzymatic oxidation of ketone bodies as glyoxal, methylglyoxal, glucosone, and 3-deoxyglucosone is not so tightly controlled [48, 49]. In general, unlike enzymatic reactions, nonenzymatic processes are not tightly controlled, and therefore they can be harmful.

**Figure 2:**
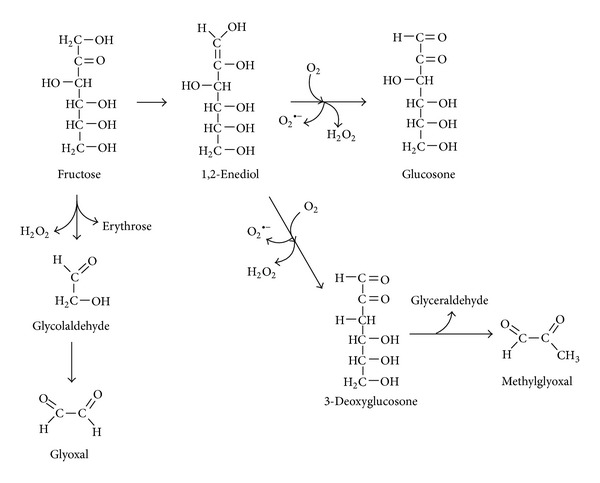
Fructose autoxidation (Wolff pathway).

**Figure 3:**
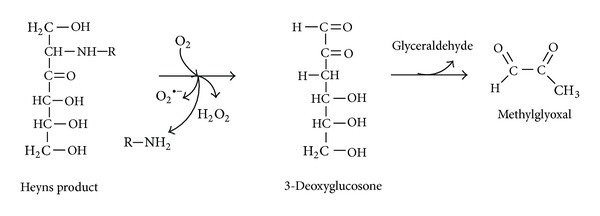
Autoxidation of Heyns compounds (Hodge pathway).

**Figure 4:**
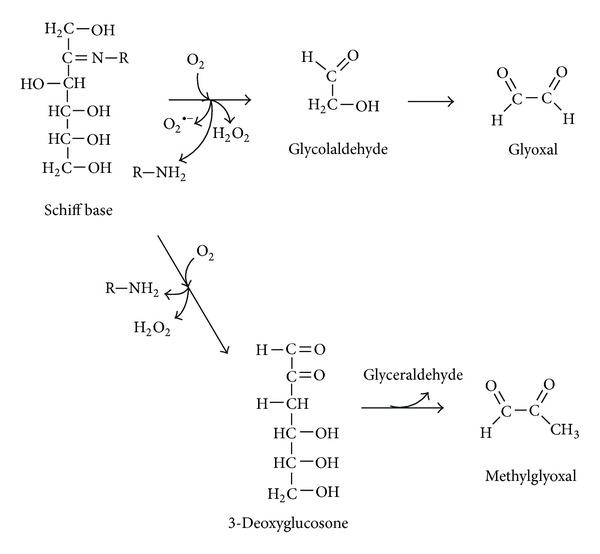
Oxidative fragmentation of Schiff base (Namiki pathway).

**Figure 5:**
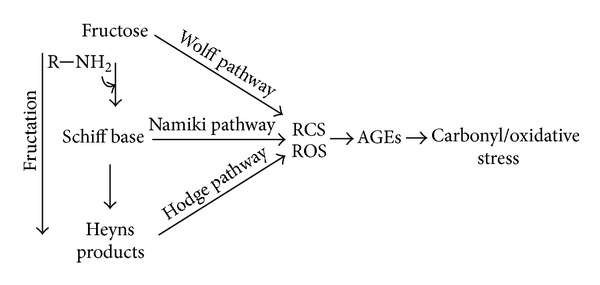
Formation of reactive species by the fructation leading to carbonyl/oxidative stress.

Oxidation reactions and ROS have been shown to be involved and frequently accelerate the fructation, glucation, and other nonenzymatic processes [24, 36]. In order to reflect the interplay between glycation and oxidative steps the “glycoxidation” term has been introduced [53]. Figure 1 shows the mechanism of fructation followed by the generation of reactive di- and tricarbonyls as well as such ROS as superoxide, hydrogen peroxide, and hydroxyl radical. Slow oxidative degradation of monosaccharides under physiological conditions also leads to the formation of *α*-dicarbonyls and some ROS. This process has been called monosaccharide autoxidation or the Wolff pathway [44, 54, 55].  Figure 2 demonstrates the mechanism of fructose autoxidation. Like other early glycation products, the Heyns compounds may undergo autoxidation (Figure 3). This process leading to the formation of RCS and ROS has been called the Hodge pathway [35, 56, 57]. In addition, there is an evidence for the fragmentation of the Schiff base that results in RCS and ROS generation (Figure 4). The series of reaction pathways in glycation established the Schiff base fragmentation to RCS and ROS now collectively called the Namiki pathway [44, 51, 58, 59]. Thus, some stages of glycoxidation demonstrate a strong relationship between carbonyl and oxidative stress (Figure 5). Interestingly, some compounds were simultaneously identified as the intermediates or end-products of glycoxidation and lipid peroxidation that confirms an interplay between both the nonenzymatic processes [49].

**Figure 6:**
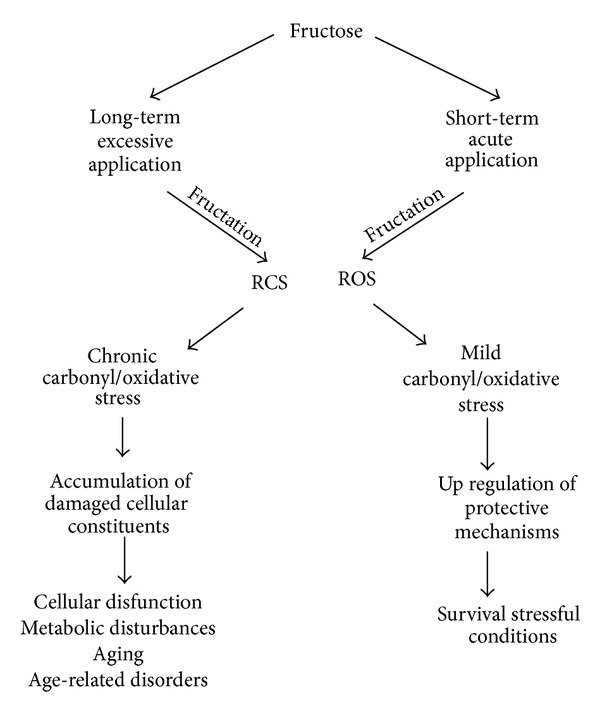
Involvement of reactive oxygen and carbonyl species in cytotoxic and defensive effects of fructose.

In the late stage of glycation, the reactive carbonyls and the Heyns compounds again interact with free amino, sulfhydryl, and guanidine functional groups of intracellular or extracellular biomolecules like proteins, nucleic acids, and aminophospholipids, resulting in their nonenzymatic, irreversible modification and formation of a variety of adducts and crosslinks collectively named advanced glycation end products (AGEs) [44, 45]. Therefore, the Heyns products and RCS formed during fructation are believed to be important precursors of nonenzymatic adduct formation in biological systems. In general, AGEs are poorly degraded complexes (Figure 6) accumulation of which increases with aging. They were detected in a variety of human tissues and serve as biomarkers of aging and age-related disorders [36]. Interestingly, the comparison of nutrition and plasma AGEs in vegetarian and omnivorous groups showed that the higher intake of fructose in alternative nutrition of healthy subjects may cause an increase of AGE levels [60]. 

It should be noted that AGEs may undergo covalent interactions with biomolecules giving more complex cross-links. In addition, AGEs are efficient *in vivo* sources of RCS and ROS [44, 51, 61–63]. Like free-radical chain reaction, glycation is characterized by unpredictable direction and a wide variety of intermediates and end-products. That is why the term “Maillard chemistry” is widely used to describe the complicity of glycation [36, 43–45, 49]. 

## 3. Adverse Side Effects of Long-Term Consumption of Fructose

Fructose is commonly used as a sweetener and its intake has quadrupled since the early 1900s, in part because of the introduction of high-fructose corn syrup [1]. This phenomenon parallels the development and progression of such disorders as obesity, type 2 diabetes mellitus and its complications, cardiovascular and neurodegenerative diseases, hypertension, and gout, liver, and kidney disease [1, 3, 7–16]. Experimental studies on animals have shown that chronic intake of excessive fructose can induce most features of the metabolic syndrome, including hypertriglyceridemia, fatty liver, nonalcoholic steatohepatitis, glucose intolerance, hyperglycemia, abdominal obesity, elevated blood pressure, microvascular disease, endothelial dysfunction, inflammation, hyperuricemia, glomerular hypertension, and renal injury [9, 16, 64–66]. 

Although the consumption of large amounts of dietary fructose can rapidly induce insulin resistance, most nutritionists believe that fructose is safer and healthier than glucose; therefore, fructose is advocated as a preferred sweetener, particularly for diabetes mellitus patients. Chronic hyperglycemia that in part can result from glucose intolerance induced by long-term consumption of fructose is a major inducer of vascular complications in diabetes (e.g., heart disease, stroke, blindness, and end-stage renal failure) which are responsible for disabilities and high mortality rates in patients with diabetes. The increased production of ROS, RCS, and AGEs as a result of glycoxidation is most preferable among the various mechanisms, which are supposed to be involved in vascular complications in diabetes. In general, the enhanced levels of glycoxidation products can explain the detrimental effects of fructose due to its long-term application in different experimental models [67–71]. 

To compare glucose and fructose involvement in the generation of glycoxidation products, recently we used baker's yeast as a model and found higher level of carbonyl/oxidative stress markers, which were correlated with a higher aging rate of fructose-grown compared with glucose-grown yeast at stationary phase (long-term model) [25, 26]. We suggested that fructose rather than glucose is more extensively involved in glycoxidation* in vivo*, yeast aging, and development of carbonyl/oxidative stress. O'Brien with colleagues found that excessive fructose intake in animal models caused tissue damage associated with carbonyl/oxidative stress [72, 73]. *In vitro* experiments also demonstrated that fructose produced greater amounts of ROS and RCS than did glucose [24, 68, 69, 74]. 

At the first glance, from the point of view of basic organic chemistry it may seem surprising, since due to a greater electrophilicity and accessibility of the carbonyl group of aldoses (e.g., glucose), their reactivity is believed to be higher than that of respective ketoses (e.g., fructose). There is some information confirming higher reactivity of glucose versus fructose in nonenzymatic processes *in vitro* [75–77], while the opposite is reported in numerous *in vitro* and *in vivo* studies [24, 74, 78–80]. Possible explanation is that glucose is less reactive due to formation of very stable ring structures in aqueous solutions (glucopyranose and glucofuranose) which retards its reactivity. Generally, glucose is the least reactive monosaccharide and this characteristic can be considered to the emergence of glucose as the primary metabolic fuel [36, 81, 82]. Fructose also forms both pyranose and furanose structures [83] but exists to a greater extent in the open-chain active form than does glucose. The proportion of acyclic forms of glucose and fructose in aqueous solution accounts for 0.001–0.002% and 0.7%, respectively [48, 84]. Thus fructose is a more potent glycoxidation agent as compared with glucose that can explain its detrimental effects.

## 4. Short-Term Application of Fructose Protects against Oxidative Stress

Although a long-term consumption of excessive fructose may have adverse side effects, its acute temporary ingestion can be beneficial under some circumstances. For example, short-term application of fructose has been found to protect astroglial C6 cells against peroxide-induced stress [21]. In contrast to glucose, fructose inhibited apoptosis induced by reoxygenation in rat hepatocytes by decreasing the level of ROS [17]. Fructose has also been found to defend rat hepatocytes against exogenous oxidative stress [18, 20]. It has been demonstrated that fructose and its phosphorylated derivatives such as fructose-1,6-bisphosphate had significantly higher antioxidant capacities against ROS than other carbohydrates [22]. Based on these phenomena, it was suggested that acute infusion or ingestion of fructose could be of benefit in the cytoprotective therapy of disorders related to oxidative stress [21]. According to homeostasis theory, the steady-state concentration of oxidants as well as antioxidants is maintained at the limited range [85]. That is why antioxidant therapy is generally found to be ineffective [30, 31]. In contrast to strong antioxidants, fructose and its phosphorylated derivatives (e.g., fructose-1,6-bisphosphate) being important energy substrates are not “fought” by redox homeostatic mechanisms. The beneficial effects of fructose-1,6-bisphosphate have been documented in different tissues, including the heart, liver, kidney, brain, and small intestine [86–89]. The cytoprotective mechanisms underlying fructose-1,6-bisphosphate are believed to be involved in its intervention in the glycolytic pathway, as a metabolic regulator or substrate, as well as an agent modifying the ion permeability of cell membrane transporters.

Recently we have demonstrated that fructose-grown yeast at exponential phase (short-term model) exposed to hydrogen peroxide demonstrated higher survival compared to glucose-grown cells [23]. In this study, significantly higher total level of ROS was observed in fructose-grown than that in glucose-grown cells under control conditions (without H_2_O_2_). However, under peroxide-induced stress ROS amount significantly decreased in yeast grown on fructose, whereas it increased in glucose-grown cells, which was very consistent with the work of Spasojević et al. [21]. The authors demonstrated a significant increase in oxidative status of astroglial C6 cells under treatment with hydrogen peroxide in glucose medium, but it was not the case in a fructose-containing medium. At the same time, hydrogen peroxide led to a decrease of C6 cell viability in both media investigated; however, the survival was higher in fructose-containing medium. In accordance with another work by Spasojević et al. [22], we demonstrated that hydrogen peroxide did not markedly change hydroxyl radical level in glucose-grown cells but it did decrease it significantly in fructose-grown cells [23]. 

An obvious question arises: what mechanism(s) is (are) responsible for the protective effect of fructose short-term application? Analysis of the literature data leads us to propose several mechanisms responsible for the defensive effect of fructose: (1) iron binding and prevention of the Fenton reaction [18, 21]; (2) stabilization of the glutathione pool in the cell [17]; (3) upregulation of the pentose phosphate pathway producing NADPH [22]; and (4) production of fructose-1,6-bisphosphate, the compound with cytoprotective and antioxidant mechanisms [86–89].

Our study extended the earlier findings with the involvement of SOD and catalase in the reduction of ROS level in fructose-grown yeast exposed to H_2_O_2_ [23]. It was shown that a reduced ROS level in fructose-grown cells was consistent with a broad peak of SOD and catalase activation by hydrogen peroxide, whereas cells grown on glucose demonstrated a sharp rise of the enzyme activities. We also found that fructose more markedly than glucose activated glyoxalases, the fundamental function of which is the metabolism of reactive *α*-dicarbonyl metabolites in most living organisms [43, 90–92]. 

These findings prompted us to propose additional explanation of fructose protective effect—a short-term application of fructose induces a mild carbonyl/oxidative stress-stimulating cellular defensive mechanisms responsible for cell survival under lethal stress [23]. The last mechanism can be posited from the *in vitro* and *in vivo* studies reporting that fructose is a much more potent glycoxidation agent, capable of producing greater amounts of RCS and ROS than glucose [21, 23–26, 68, 74]. Generally, ROS and RCS are found to play a dual role *in vivo*, which appears to be concentration dependent [23, 27–32]. At high concentrations, reactive species are potentially dangerous, as they can cause damage to cell constituents that, in turn, accompany aging and age-related disorders. In contrast, the beneficial effects of ROS and RCS occur at low concentrations and involve physiological roles in cellular signaling pathways, responses to environmental challenges, and so forth. It is also well documented that mild stress caused by low doses of reactive species can result in the acquisition of cellular resistance to lethal stress [28, 93–96].

## 5. Conclusion

Considering the literature data it can be supposed that at long-term consumption of excessive fructose chronic increase in the levels of reactive species leads to the accumulation of damaged cellular constituents resulting in cellular disfunction, whereas short-term application of fructose provokes a mild stress, resulting in the acquisition of cellular cross-resistance to lethal stress (Figure 6). Therefore, we suggest the involvement of reactive species like RCS and ROS in both the cytotoxic and defensive effects of fructose. Thus, depending on conditions, fructose can play a dual role in the living cell. Long-term application of excessive fructose leads to glycoxidation, generation of ROS and RCS, and accumulation of damaged cellular constituents which is suggested to accompany the aging process, cellular dysfunction. and age-related disorders. In opposite, short-term application of fructose provokes a mild carbonyl/oxidative stress, resulting in the acquisition of cellular cross-resistance to lethal stress. Generally, a long-term consumption of excessive fructose is found to have adverse side effects; however acute temporary application of fructose can be beneficial under some pathophysiological conditions. 

## References

[1] White JS (2013). Challenging the fructose hypothesis: new perspectives on fructose consumption and metabolism. *Advances in Nutrition*.

[2] Rippe JM, Angelopoulos TJ (2013). Sucrose, high-fructose corn syrup, and fructose, their metabolism and potential health effects: what do we really know?. *Advances in Nutrition*.

[3] Bray GA (2013). Energy and fructose from beverages sweetened with sugar or high-fructose corn syrup pose a health risk for some people. *Advances in Nutrition*.

[4] Karalius VP, Shoham DA (2013). Dietary sugar and artificial sweetener intake and chronic kidney disease: a review. *Advances in Chronic Kidney Disease*.

[5] Dekker MJ, Su Q, Baker C, Rutledge AC, Adeli K (2010). Fructose: a highly lipogenic nutrient implicated in insulin resistance, hepatic steatosis, and the metabolic syndrome. *The American Journal of Physiology—Endocrinology and Metabolism*.

[6] Tappy L (2012). Q&A: “toxic” effects of sugar: should we be afraid of fructose?. *BMC Biology*.

[7] Gaby AR (2005). Adverse effects of dietary fructose. *Alternative Medicine Review*.

[8] Johnson RJ, Segal MS, Sautin Y (2007). Potential role of sugar (fructose) in the epidemic of hypertension, obesity and the metabolic syndrome, diabetes, kidney disease, and cardiovascular disease. *The American Journal of Clinical Nutrition*.

[9] Sánchez-Lozada LG, Le M, Segal M, Johnson RJ (2008). How safe is fructose for persons with or without diabetes?. *The American Journal of Clinical Nutrition*.

[10] Stanhope KL, Schwarz JM, Havel PJ (2013). Adverse metabolic effects of dietary fructose: results from the recent epidemiological, clinical, and mechanistic studies. *Current Opinion in Lipidology*.

[11] Stanhope KL, Schwarz JM, Keim NL (2009). Consuming fructose-sweetened, not glucose-sweetened, beverages increases visceral adiposity and lipids and decreases insulin sensitivity in overweight/obese humans. *Journal of Clinical Investigation*.

[12] Tappy L, Lê KA, Tran C, Paquot N (2010). Fructose and metabolic diseases: new findings, new questions. *Nutrition*.

[13] Tappy L, Lê K (2010). Metabolic effects of fructose and the worldwide increase in obesity. *Physiological Reviews*.

[14] Lanaspa MA, Tapia E, Soto V, Sautin Y, Sánchez-Lozada LG (2011). Uric acid and fructose: potential biological mechanisms. *Seminars in Nephrology*.

[15] Soleimani M (2011). Dietary fructose, salt absorption and hypertension in metabolic syndrome: towards a new paradigm. *Acta Physiologica*.

[16] Rebollo A, Roglans N, Alegret M, Laguna JC (2012). Way back for fructose and liver metabolism: bench side to molecular insights. *World Journal of Gastroenterology*.

[17] Frenzel J, Richter J, Eschrich K (2002). Fructose inhibits apoptosis induced by reoxygenation in rat hepatocytes by decreasing reactive oxygen species via stabilization of the glutathione pool. *Biochimica et Biophysica Acta*.

[18] Valeri F, Boess F, Wolf A, Göldlin C, Boelsterli UA (1996). Fructose and tagatose protect against oxidative cell injury by iron chelation. *Free Radical Biology and Medicine*.

[19] Bogdanović J, Mojović M, Milosavić N, Mitrović A, Vucinić Z, Spasojević I (2008). Role of fructose in the adaptation of plants to cold-induced oxidative stress. *European Biophysics Journal*.

[20] MacAllister SL, Choi J, Dedina L, O’Brien PJ (2011). Metabolic mechanisms of methanol/formaldehyde in isolated rat hepatocytes: carbonyl-metabolizing enzymes versus oxidative stress. *Chemico-Biological Interactions*.

[21] Spasojević I, Bajić A, Jovanović K, Spasić M, Andjus P (2009). Protective role of fructose in the metabolism of astroglial C6 cells exposed to hydrogen peroxide. *Carbohydrate Research*.

[22] Spasojević I, Mojović M, Blagojević D (2009). Relevance of the capacity of phosphorylated fructose to scavenge the hydroxyl radical. *Carbohydrate Research*.

[23] Semchyshyn HM, Lozinska LM (2012). Fructose protects baker's yeast against peroxide stress: potential role of catalase and superoxide dismutase. *FEMS Yeast Research*.

[24] Sakai M, Oimomi M, Kasuga M (2002). Experimental studies on the role of fructose in the development of diabetic complications. *Kobe Journal of Medical Sciences*.

[25] Lozinska LM, Semchyshyn GM (2011). Fructose as a factor of carbonyl and oxidative stress development and accelerated aging in the yeast *Saccharomyces cerevisiae*. *Ukrainian Biochemical Journal*.

[26] Semchyshyn HM, Lozinska LM, Miedzobrodzki J, Lushchak VI (2011). Fructose and glucose differentially affect aging and carbonyl/oxidative stress parameters in *Saccharomyces cerevisiae* cells. *Carbohydrate Research*.

[27] Mesquita A, Weinberger M, Silva A (2010). Caloric restriction or catalase inactivation extends yeast chronological lifespan by inducing H_2_O_2_ and superoxide dismutase activity. *Proceedings of the National Academy of Sciences of the United States of America*.

[28] Semchyshyn H (2009). Hydrogen peroxide-induced response in *E. coli* and *S. cerevisiae*: different stages of the flow of the genetic information. *Central European Journal of Biology*.

[29] Forman HJ (2010). Reactive oxygen species and *α*,*β*-unsaturated aldehydes as second messengers in signal transduction. *Annals of the New York Academy of Sciences*.

[30] Spasojević I, Jones DR, Andrades ME (2012). Hydrogen peroxide in adaptation. *Oxidative Medicine and Cellular Longevity*.

[31] Brieger K, Schiavone S, Miller FJ, Krause KH (2012). Reactive oxygen species: from health to disease. *Swiss Medical Weekly*.

[32] Pohanka M (2013). Role of oxidative stress in infectious diseases. A review. *Folia Microbiologica*.

[33] Maillard LC (1912). Action des acides aminés sur les sucres: formation des mélanoïdines par voie méthodique. *Comptes Rendus de l'Academie des Sciences*.

[34] Maillard LC (1912). Formation d’humus et de combustibles mineraux sans intervention de l’oxygene atmospherique des microorganismes, des hautes temperatures, ou des fortes pressions. *Comptes Rendus de l'Academie des Sciences*.

[35] Hodge JE (1953). Dehydrated foods: chemistry of browning reactions in model systems. *Journal of Agricultural and Food Chemistry*.

[36] Tessier FJ (2010). The Maillard reaction in the human body. The main discoveries and factors that affect glycation. *Pathologie Biologie*.

[37] Rahbar S (1968). Hemoglobin H disease in two Iranian families. *Clinica Chimica Acta*.

[38] Rahbar S, Blumenfeld O, Ranney HM (1969). Studies of an unusual hemoglobin in patients with diabetes mellitus. *Biochemical and Biophysical Research Communications*.

[39] Yatscoff RW, Tevaarwerk GJ, MacDonald JC (1984). Quantification of nonenzymically glycated albumin and total serum protein by affinity chromatography. *Clinical Chemistry*.

[40] Monnier VM, Cerami A (1981). Nonenzymatic browning *in vivo*: possible process for aging of long-lived proteins. *Science*.

[41] Cerami A (1985). Hypothesis: glucose as a mediator of aging. *Journal of the American Geriatrics Society*.

[42] Finot PA (1982). Nonenzymatic browning products: physiologic effects and metabolic transit in relation to chemical structure. A review. *Diabetes*.

[43] Ellis EM (2007). Reactive carbonyls and oxidative stress: potential for therapeutic intervention. *Pharmacology and Therapeutics*.

[44] Peng X, Ma J, Chen F, Wang M (2011). Naturally occurring inhibitors against the formation of advanced glycation end-products. *Food and Function*.

[45] Robert L, Robert AM, Labat-Robert J (2011). The Maillard reaction—illicite (bio)chemistry in tissues and food. *Pathologie Biologie*.

[46] Miyata T, van Ypersele de Strihou C, Kurokawa K, Baynes JW (1999). Alterations in nonenzymatic biochemistry in uremia: origin and significance of “carbonyl stress” in long-term uremic complications. *Kidney International*.

[47] Moheimani F, Morgan PE, van Reyk DM, Davies MJ (2010). Deleterious effects of reactive aldehydes and glycated proteins on macrophage proteasomal function: possible links between diabetes and atherosclerosis. *Biochimica et Biophysica Acta*.

[48] Turk Z (2010). Glycotoxines, carbonyl stress and relevance to diabetes and its complications. *Physiological Research*.

[49] Semchyshyn HM, Lushchak VI, Lushchak VI, Semchyshyn HM (2012). Interplay between oxidative and carbonyl stresses: molecular mechanisms, biological effects and therapeutic strategies of protection. *Oxidative Stress—Molecular Mechanisms and Biological Effects*.

[50] Reihl O, Rothenbacher TM, Lederer MO, Schwack W (2004). Carbohydrate carbonyl mobility—the key process in the formation of *α*-dicarbonyl intermediates. *Carbohydrate Research*.

[51] Thornalley PJ (2005). Dicarbonyl intermediates in the Maillard reaction. *Annals of the New York Academy of Sciences*.

[52] Smuda M, Glomb MA (2011). Novel insights into the Maillard catalyzed degradation of maltose. *Journal of Agricultural and Food Chemistry*.

[53] Dyer DG, Blackledge JA, Katz BM (1991). The Maillard reaction *in vivo*. *Zeitschrift fur Ernahrungswissenschaft*.

[54] Thornalley PJ, Wolff SP, Crabbe J, Stern A (1984). The autoxidation of glyceraldehyde and other simple monosaccharides under physiological conditions catalysed by buffer ions. *Biochimica et Biophysica Acta*.

[55] Wolff SP, Jiang ZY, Hunt JV (1991). Protein glycation and oxidative stress in diabetes mellitus and ageing. *Free Radical Biology and Medicine*.

[56] Hunt JV, Dean RT, Wolff SP (1988). Hydroxyl radical production and autoxidative glycosylation. Glucose autoxidation as the cause of protein damage in the experimental glycation model of diabetes mellitus and ageing. *Biochemical Journal*.

[57] Mullarkey CJ, Edelstein D, Brownlee M (1990). Free radical generation by early glycation products: a mechanism for accelerated atherogenesis in diabetes. *Biochemical and Biophysical Research Communications*.

[58] Hayashi T, Namiki M (1980). Formation of two-carbon sugar fragments at an early stage of the browning reaction of sugar and amine. *Agricultural Biology and Chemistry*.

[59] Namiki M, Hayashi T, Waller GR, Feather MS (1983). A new mechanism of the Maillard reaction involving sugar fragmentation and free radical formation. *The Maillard Reaction in Foods and Nutrition*.

[60] Krajcovicová-Kudlácková M, Sebeková K, Schinzel R, Klvanová J (2002). Advanced glycation end products and nutrition. *Physiological Reviwes*.

[61] Yim MB, Yim HS, Lee C, Kang SO, Chock PB (2001). Protein glycation: creation of catalytic sites for free radical generation. *Annals of the New York Academy of Sciences*.

[62] Takamiya R, Takahashi M, Myint T (2003). Glycation proceeds faster in mutated Cu, Zn-superoxide dismutases related to familial amyotrophic lateral sclerosis. *The FASEB Journal*.

[63] Shumaev KB, Gubkina SA, Kumskova EM, Shepelkova GS, Ruuge EK, Lankin VZ (2009). Superoxide formation as a result of interaction of L-lysine with dicarbonyl compounds and its possible mechanism. *Biochemistry*.

[64] Alegret M, Roglans N, Laguna JC, Hemling RM, Belkin AT (2011). Fructose consumption and leptin resistance: what have we learnt from animal studies?. *Leptin: Hormonal Functions, Dysfunctions and Clinical Uses*.

[65] Vilà L, Roglans N, Perna V (2011). Liver AMP/ATP ratio and fructokinase expression are related to gender differences in AMPK activity and glucose intolerance in rats ingesting liquid fructose. *Journal of Nutritional Biochemistry*.

[66] Cummings BP, Stanhope KL, Graham JL (2010). Dietary fructose accelerates the development of diabetes in UCD-T2DM rats: amelioration by the antioxidant, *α*-lipoic acid. *The American Journal of Physiology—Regulatory Integrative and Comparative Physiology*.

[67] Levi B, Werman MJ (1998). Long-term fructose consumption accelerates glycation and several age-related variables in male rats. *Journal of Nutrition*.

[68] Levi B, Werman MJ (2001). Fructose triggers DNA modification and damage in an *Escherichia coli* plasmid. *Journal of Nutritional Biochemistry*.

[69] Levi B, Werman MJ (2003). Fructose and related phosphate derivatives impose DNA damage and apoptosis in L5178Y mouse lymphoma cells. *Journal of Nutritional Biochemistry*.

[70] Lee O, Bruce WR, Dong Q, Bruce J, Mehta R, O’Brien PJ (2009). Fructose and carbonyl metabolites as endogenous toxins. *Chemico-Biological Interactions*.

[71] Lushchak OV, Rovenko BM, Gospodaryov DV, Lushchak VI (2011). Drosophila melanogaster larvae fed by glucose and fructose demonstrate difference in oxidative stress markers and antioxidant enzymes of adult flies. *Comparative Biochemistry and Physiology*.

[72] Dong Q, Yang K, Wong SM, O’Brien PJ (2010). Hepatocyte or serum albumin protein carbonylation by oxidized fructose metabolites: glyceraldehyde or glycolaldehyde as endogenous toxins?. *Chemico-Biological Interactions*.

[73] Yang K, Feng C, Lip H, Bruce WR, O’Brien PJ (2011). Cytotoxic molecular mechanisms and cytoprotection by enzymic metabolism or autoxidation for glyceraldehyde, hydroxypyruvate and glycolaldehyde. *Chemico-Biological Interactions*.

[74] Suarez G, Rajaram R, Oronsky AL, Gawinowicz MA (1989). Nonenzymatic glycation of bovine serum albumin by fructose (fructation). Comparison with the Maillard reaction initiated by glucose. *The Journal of Biological Chemistry*.

[75] Wijewickreme AN, Kitts DD, Durance TD (1997). Reaction conditions influence the elementary composition and metal chelating affinity of nondialyzable model Maillard reaction products. *Journal of Agricultural and Food Chemistry*.

[76] Yeboah FK, Alli I, Yaylayan VA (1999). Reactivities of D-glucose and D-fructose during glycation of bovine serum albumin. *Journal of Agricultural and Food Chemistry*.

[77] Rangsansarid J, Cheetangdee N, Kinoshita N, Fukuda K (2008). Bovine serum albumin-sugar conjugates through the Maillard reaction: effects on interfacial behavior and emulsifying ability. *Journal of Oleo Science*.

[78] Suarez G, Maturana J, Oronsky AL, Raventos-Suarez C (1991). Fructose-induced fluorescence generation of reductively methylated glycated bovine serum albumin: evidence for nonenzymatic glycation of Amadori adducts. *Biochimica et Biophysica Acta*.

[79] Robert L, Labat-Robert J, Robert AM (2010). The Maillard reaction. From nutritional problems to preventive medicine. *Pathologie Biologie*.

[80] Lawrence GD, Mavi A, Meral K (2008). Promotion by phosphate of Fe(III)- and Cu(II)-catalyzed autoxidation of fructose. *Carbohydrate Research*.

[81] Bunn HF, Higgins PJ (1981). Reaction of monosaccharides with proteins: possible evolutionary significance. *Science*.

[82] Dills WL (1993). Protein fructosylation: fructose and the Maillard reaction. *The American Journal of Clinical Nutrition*.

[83] Cocinero EJ, Lesarri A, Ecija P (2013). Free fructose is conformationally locked. *Journal of the American Chemical Society*.

[84] Hayward LD, Angyal SJ (1977). A symmetry rule for the circular dichroism of reducing sugars, and the proportion of carbonyl forms in aqueous solutions thereof. *Carbohydrate Research*.

[85] Lushchak VI (2011). Adaptive response to oxidative stress: bacteria, fungi, plants and animals. *Comparative Biochemistry and Physiology C*.

[86] Muntané J (2006). Multiple beneficial actions of fructose 1,6-biphosphate in sepsis-associated liver injury. *Critical Care Medicine*.

[87] Marangos PJ, Fox AW, Riedel BJ, Royston D, Dziewanowska ZE (1998). Potential therapeutic applications of fructose-1,6-diphosphate. *Expert Opinion on Investigational Drugs*.

[88] Lopes RP, Lunardelli A, Preissler T (2006). The effects of fructose-1,6-bisphosphate and dexamethasone on acute inflammation and T-cell proliferation. *Inflammation Research*.

[89] Riedel BJ, Gal J, Ellis G, Marangos PJ, Fox AW, Royston D (2004). Myocardial protection using fructose-1,6-diphosphate during coronary artery bypass graft surgery: a randomized, placebo-controlled clinical trial. *Anesthesia and Analgesia*.

[90] Thornalley PJ (1990). The glyoxalase system: new developments towards functional characterization of a metabolic pathway fundamental to biological life. *Biochemical Journal*.

[91] Inoue Y, Maeta K, Nomura W (2011). Glyoxalase system in yeasts: structure, function, and physiology. *Seminars in Cell and Developmental Biology*.

[92] Xue M, Rabbani N, Thornalley PJ (2011). Glyoxalase in ageing. *Seminars in Cell and Developmental Biology*.

[93] Collinson LP, Dawes IW (1992). Inducibility of the response of yeast cells to peroxide stress. *Journal of General Microbiology*.

[94] Bayliak MM, Semchyshyn HM, Lushchak VI (2007). Possible accumulation of non-active molecules of catalase and superoxide dismutase in *S. cerevisiae* cells under hydrogen peroxide induced stress. *Central European Journal of Biology*.

[95] Semchyshyn HM, Abrat OB, Miedzobrodzki J, Inoue Y, Lushchak VI (2011). Acetate but not propionate induces oxidative stress in bakers’ yeast *Saccharomyces cerevisiae*. *Redox Report*.

[96] Berry DB, Gasch AP (2008). Stress-activated genomic expression changes serve a preparative role for impending stress in yeast. *Molecular Biology of the Cell*.

